# Copolymerization Behavior of Acrylamide-Based Polymers in Ionic Liquid Media

**DOI:** 10.3390/polym17141963

**Published:** 2025-07-17

**Authors:** Gaoshen Su, Jingyi Cui, Chaoyang Li, Ping Chen, Yong Li, Wenxue Jiang, Huan Yang, Xiaorong Yu, Liangliang Wang

**Affiliations:** 1 College of Chemistry and Environmental Engineering, Yangtze University, Jingzhou 434023, China; cuijingyi628@163.com (J.C.); chaoyangli1998@163.com (C.L.); yanghuan@yangtzeu.edu.cn (H.Y.); yxr_cjdx@163.com (X.Y.); 2 Drilling & Production Engineering Technology Research Institute, CNPC Chuanqing Drilling Engineering Company Limited, Xi’an 710018, China; chen_ping@cnpc.com.cn (P.C.); liyong_gcy@cnpc.com.cn (Y.L.); wenxue_j@cnpc.com.cn (W.J.); 3 College of Petroleum Engineering, China University of Petroleum, Qingdao 266000, China

**Keywords:** ionic liquid, reaction medium, acrylamide copolymer, copolymer properties

## Abstract

To examine how reaction media influence the copolymerization processes of acrylamide-based copolymers, [BMIM]Oac and water were utilized as the reaction media. Four copolymers P(AM-SSS) (H_2_O), P(AM-UA) (H_2_O), P(AM-SSS) (ILs), and P(AM-UA) (ILs) were synthesized using the soluble monomer sodium p-styrene sulfonate (SSS), the insoluble monomer 10-undecylenoic acid (UA), and acrylamide (AM). The properties of the copolymers were characterized using infrared spectroscopy and ^1^H NMR, and the copolymerization rates of the monomers and the segment sequences of the copolymers were calculated. The results indicated that copolymerization of SSS in ionic liquids could reduce the length of the continuous units of AM in the copolymer’s molecular chain from 231.2866 to 91.1179, with a more uniform distribution within the molecular chain. The thermal stability and micro-morphology of the copolymers were tested using a synchronous thermal analyzer and scanning electron microscopy, and the resistance of the copolymer solutions to temperature, salt, and shear were evaluated. Comparisons revealed that the three-dimensional spatial structure formed by the copolymers in ionic liquids is robust and loose. When AM and SSS polymerize in [BMIM]Oac, the resulting copolymer exhibits a higher viscosity retention rate in temperature and shear resistance tests, with a thermal decomposition temperature reaching 260 °C. Conversely, when AM and UA polymerize in [BMIM]Oac, the copolymer demonstrates good salt resistance, maintaining a viscosity retention rate of 259.04% at a Na^+^ concentration of 200,000 mg/L. Therefore, the ionic liquid [BMIM]Oac can enhance the various application performances of copolymers formed by monomers with different solubilities and AM.

## 1. Introduction

During the continuous development of oil and gas resources, approximately two-thirds of the original oil remains in the reservoir after traditional primary (POR) and secondary (SOR) extraction [1]. Polymer flooding, a widely used technique for enhancing crude oil production, has seen the extensive use of acrylamide (AM) copolymers, particularly in major oil fields in China, due to their favorable performance [2,3]. Recently, hydrophobic association polymers with a small number of hydrophobic monomers have attracted a lot of attention due to their excellent rheological properties. These polymers demonstrate remarkable compatibility with salts and surfactants, making them valuable materials for further research [4,5,6]. However, hydrophobic monomers generally exhibit poor water solubility compared to traditional water-soluble copolymerization monomers, posing a challenge for their incorporation into polymer chains via conventional aqueous solution polymerization methods [7,8]. To enhance the solubility of hydrophobic monomers and optimize copolymerization efficiency, micellar polymerization has emerged as a promising approach [9]. For instance, Fan et al. [10] employed sodium dodecyl sulfate (SDS) as a micellar agent in aqueous solution to facilitate the copolymerization of 2-acrylamido-2-methylpropanesulfonic acid (AMPS), octadecyl dimethyl allyl ammonium chloride (DMAAC-18), and octadecyl methacrylate (SMA) with acrylamide (AM). Subsequent hydrolysis yielded a hydrophobic associative copolymer (HPAOS) capable of synergistic interactions with monomers. Performance evaluations confirmed the polymer’s excellent thermal stability and salt tolerance. Similarly, J. G. Torres-Martinez [11] et al. demonstrated that the addition of SDS to the polymerization system for micellar copolymerization significantly improves the performance of the polymers for applications. Nevertheless, AM homopolymerization is the main polymerization method in the later stage of micellar polymerization [12], which may lead to serious inhomogeneity of the microstructure and also have a great impact on the viscosity of the copolymer [13,14,15]. Furthermore, the surfactants used in the polymerization process are difficult to remove from the final products, potentially affecting their overall quality and performance [16].

Ionic liquids (ILs) have emerged as versatile solvents in polymer science owing to their customizable physicochemical properties through cation/anion structural modifications [17]. Researchers have utilized ionic liquids as reaction media for various polymerization reactions, such as free-radical polymerization, ionic polymerization, and condensation polymerization, discovering that the resulting polymers possess high molecular weights, high polymerization efficiency, and stronger stability [18,19]. Liang [20] conducted free-radical polymerization using acrylonitrile (AN) in [BMIM]BF_4_, indicating that ionic liquids can serve as excellent solvents for obtaining high-molecular-weight polymers. It was explained that ionic liquids have low chain transfer constants, which can stabilize active free radicals during the polymerization process. Compared to traditional solvents, the thermal stability of polymers synthesized in ionic liquids is significantly enhanced. Kubisa [21] explained in his study on polymerization kinetics that the effect of ionic liquids as reaction media on polymerization reactions is generally associated with the higher viscosity of ionic liquids (primarily affecting termination) and the specific interactions between the growing free radicals and the components of the ionic liquids (primarily affecting propagation). In a separate study, Liu et al. [22] incorporated a polymerizable IL (MMIB) into P(AM/AA/MMIB/NIEA) copolymers, with characterization data confirming enhanced solubility and rheological performance relative to traditional hydrophobic associative polymers. These improvements are attributed to hydrophilic ILs’ capacity to increase solution polarity, which positively influences flow behavior [23,24].

Currently, there are relatively few studies examining the use of ILs as reaction media for AM-based copolymers [8,25,26]. In this study, copolymerization was conducted using different solubility copolymer monomers (sodium p-styrene sulfonate and 10-undecenoic acid) with acrylamide (AM) in both aqueous solution and [BMIM]Oac. The copolymerization rates of the monomers were calculated, and the properties of the copolymer products were tested to investigate the effect of the ionic liquid [BMIM]Oac as a reaction medium on acrylamide-based polymers. By combining the unique properties of ionic liquids (ILs) with the performance of acrylamide-based polymers, this research aims to explore new types of polymer materials and provide more possibilities for the preparation processes of acrylamide-based polymers and the potential applications of ILs.

## 2. Materials and Methods

### 2.1. Materials

1-butyl-3-methylimidazolium acetate ([BMIM]Oac, 99%) was purchased from Suzhou Senfeida Chemical Co., Ltd. (Suzhou, China). Sodium p-styrene sulfonate (SSS, ≥90%), Acrylamide (AM, 99%), 10-undecylenoic acid (UA, ≥98%), and Sodium dodecyl Sulfate (SDS, ≥98%) were purchased from Aladdin Biochemical Technology Co., Ltd. (Shanghai, China). Methanol and anhydrous ethanol were purchased from Zhiyuan Chemical Reagent Co., Ltd. (Tianjin, China). The initiator was laboratory synthesized. All chemicals were used in their as-received state without undergoing any additional purification procedures.

### 2.2. Equipment

The following instruments were employed in this study: a field emission scanning electron microscope (MIRA3, TESCAN, Shanghai, China), a simultaneous thermal analyzer (Labsys evo, SETARAM, Lyon, France), and a viscosimeter (DV Next, AMETEK Brookfield, Shanghai, China).

### 2.3. Preparation of Copolymers

**Figure 1:**
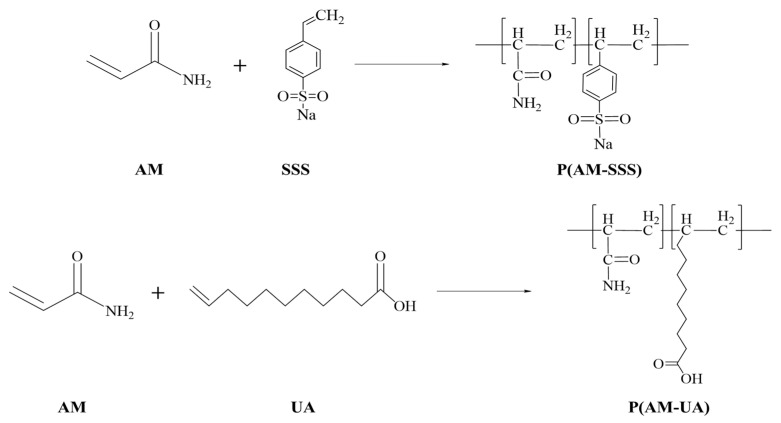
Possible synthesis reactions of copolymers.

Drawing on the prior research of our group to synthesize copolymers [6], a 50 mL three-necked flask was employed. The flask was purged with nitrogen for 10 min to expel air. Subsequently, 20 mL of [BMIM]Oac or H_2_O (with an appropriate amount of SDS added when necessary) was introduced to establish a monomer concentration of 30%. AM and comonomers (SSS and UA) were added at a mass ratio of 100:1 (copolymers with varying monomer molar ratios were synthesized for reactivity ratio determination, with specific quantities detailed in Table 1). The total amount of monomers constituted 0.8‰ of the initiator, which was added at 5 °C. Nitrogen gas was continuously maintained throughout the reaction process. After 6 h of reaction, a few drops of methanol were introduced to terminate the reaction. The resulting copolymer was successively washed with deionized water and anhydrous ethanol, followed by purification, drying, pulverization, and storage for subsequent use. The copolymers synthesized in aqueous solution were designated as P(AM-SSS) (H_2_O) and P(AM-UA) (H_2_O), while those synthesized in the ionic liquid [BMIM]Oac were labeled as P(AM-SSS) (ILs) and P(AM-UA) (ILs). The plausible reactions involved in the polymerization are depicted in Figure 1.

### 2.4. FTIR and ^1^H NMR Characterization

Structural characterization of the copolymer was performed using a Nicolet 6700 FTIR spectrometer (Thermo Fisher Scientific, Waltham, MA, USA), with spectral data collected in the range of 4000–400 cm^−1^ at a resolution of 4 cm^−1^.

Proton nuclear magnetic resonance (^1^H NMR) analysis was conducted on a Bruker AVANCE III HD 400 MHz spectrometer using deuterated water (D_2_O) as the solvent.

### 2.5. Calculation of Monomer Copolymerization Rate and Chain Segment Sequence of Copolymers

The elemental composition of carbon (C), hydrogen (H), nitrogen (N), and oxygen (O) in P(AM-SSS) and P(AM-UA) copolymers was quantitatively determined using a Vario EI Type III elemental analyzer. Based on these measurements, the contents of AM, UA, and SSS units within the copolymer were calculated. The calculation methods employed are the intercept–slope method (Fineman–Ross method, abbreviated as F-R method) as shown in Equation (1) and the line intersection method (Mayo–Lewis method, abbreviated as M-L method) as illustrated in Equation (2) [21,22].

The composition of the binary copolymer was determined by the combination of monomer composition and reactivity. Based on the monomer composition and reactivity, the copolymerization behavior of the copolymerization reaction can be ascertained, and the composition of the copolymer can be inferred. The calculation method is as shown in Equation (3).

(1) $$f_{1}-\frac{f_{1}}{F_{1}}=r_{1}\times \frac{f_{1}^{2}}{F_{1}}-r_{2}$$

(2) $$r_{2}=r_{1}\times \frac{f_{1}^{2}}{F_{1}}+\frac{f_{1}}{F_{1}}-f_{1}$$

(3) $$F_{1}=\frac{{r_{1}f}_{1}^{2}+f_{1}f_{2}}{{r_{1}f}_{1}^{2}+{2f}_{1}f_{2}+{r_{2}f}_{2}^{2}}$$

where in Equations (1)–(3), *F* represents the molar ratio of a single monomer unit to the overall units in the copolymer, while *f* signifies the molar ratio of a single monomer relative to all monomers present prior to polymerization. Additionally, *r* denotes the reactivity ratio of the monomers involved.

### 2.6. Characterization of Microscopic Morphology

The copolymer solution underwent lyophilization at −70 °C, followed by sputter-coating with gold to improve surface conductivity and imaging contrast. Morphological characterization was performed using a MIRA3-LM field emission scanning electron microscope (FESEM), enabling the comparative analysis of copolymer microstructures under two different conditions at consistent magnification.

### 2.7. Thermogravimetric Characterization (TGA)

The copolymers P(AM-SSS) (H_2_O), P(AM-UA) (H_2_O), P(AM-SSS) (ILs), and P(AM-UA) (ILs) were subjected to thermal analysis using a DSC300 thermogravimetric analyzer over a temperature range of 30 to 600 °C. The temperature increase rate was set at 10 °C/min. Thermogravimetric (TG) and derivative thermogravimetric (DTG) curves were generated to assess the thermal stability of these copolymers.

### 2.8. Viscosity Increase Performance Testing

Aqueous solutions of P(AM-SSS) (H_2_O), P(AM-UA) (H_2_O), P(AM-SSS) (ILs), P(AM-UA) (ILs), and commercial KYPAM-6S were prepared with varying concentrations at 30 °C. Rheological measurements were performed at a shear rate of 7.82 s^−1^ using a Brookfield DV-3T viscometer to determine the apparent viscosity of each polymer system.

### 2.9. Temperature and Shear Resistance Testing

For viscosity matching across five polymer systems, KYPAM-6S was prepared at 2 g/L, while ionic liquid-synthesized P(AM-SSS) (ILs) and P(AM-UA) (ILs) solutions were formulated at 12, 15, and 10 g/L in [BMIM]Oac. Corresponding aqueous-synthesized P(AM-SSS) (H_2_O) and P(AM-UA) (H_2_O) solutions were prepared at 14, 16, and 10 g/L.

Thermal stability assessment involved viscosity measurements (Brookfield DV-3T viscometer) across 30–90 °C at a 7.82 s^−1^ shear rate. Shear resistance was similarly evaluated at 30 °C with shear rates varying from 7.82 to 85 s^−1^.

### 2.10. Salt Resistance Performance Testing

The copolymers’ resistance to monovalent and divalent cations were evaluated using sodium chloride (NaCl) and calcium chloride (CaCl_2_) solutions, respectively. The concentrations of the NaCl solutions tested were 1000, 4000, 6000, 10,000, 20,000, 30,000, 40,000, 60,000, 80,000, 100,000, and 200,000 mg/L, while the concentrations of the CaCl_2_ solutions ranged from 200 to 10,000 mg/L in increments of 400. Various concentrations of these salt solutions were employed to prepare P(AM-UA) (ILs), P(AM-SSS) (ILs), P(AM-UA) (H_2_O), and P(AM-SSS) (H_2_O). The salt tolerance of the copolymers was assessed by measuring the viscosity of the solutions at 30 °C using a Brookfield DV-3T viscometer, with P(AM-UA) (H_2_O) and P(AM-SSS) (H_2_O) serving as the test samples [27].

## 3. Results

### 3.1. Characterization of Copolymers in Ils

#### 3.1.1. FTIR Analysis

**Figure 2:**
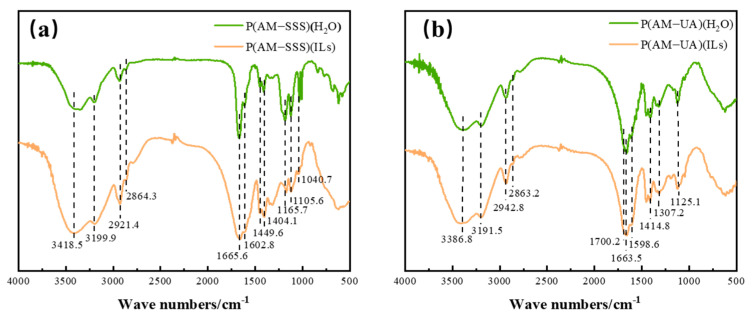
Infrared spectra of copolymers: (**a**) P(AM-SSS) (H_2_O) and P(AM-SSS) (ILs); and (**b**) P(AM-UA) (H_2_O) and P(AM-UA) (ILs).

The infrared spectra of the four copolymers, namely P(AM-SSS) (H_2_O), P(AM-UA) (H_2_O), P(AM-SSS) (ILs), and P(AM-UA) (ILs), are depicted in Figure 2. Among these copolymers, absorption peaks detected at 3418.5 cm^−1^, 3386.8 cm^−1^, and 3199.9 cm^−1^ correspond to the signature amide group N-H. Additionally, the peaks located at 1663.5 cm^−1^ and 1665.6 cm^−1^ relate to the amide group’s C=O, whereas the peak appearing at 1598.6 cm^−1^ signifies N-H bending vibrations within the amide structure. Moreover, the peak identified at 1404.1 cm^−1^ illustrates the stretching vibration of the -C-N bond in the amide group, thereby affirming the existence of AM units within the P(AM-SSS) copolymer. The distinguishing absorption peak for the benzene ring skeleton can be found at 1602.8 cm^−1^, while the peaks at 1165.7 cm^−1^, 1108.6 cm^−1^, and 1040.7 cm^−1^ are linked to the sulfonic acid group indicating that SSS units are present in the P(AM-SSS) copolymer [28]. As for the P(AM-UA) copolymer, the absorption peak for the stretching vibration of carboxylic acid C=O is observed at 1700.2 cm^−1^ along with another peak at 1307.2 cm^−1^ which corresponds to the C-O stretching vibration in carboxylic acid, confirming the presence of both AM and UA units [29]. As for the P(AM-UA) copolymer, the absorption peak for the stretching vibration of carboxylic acid C=O is observed at 1700.2 cm^−1^ along with another peak at 1307.2 cm^−1^ which corresponds to the C-O stretching vibration in carboxylic acid, confirming the presence of both AM and UA units.

#### 3.1.2. ^1^H NMR Analysis

**Figure 3:**
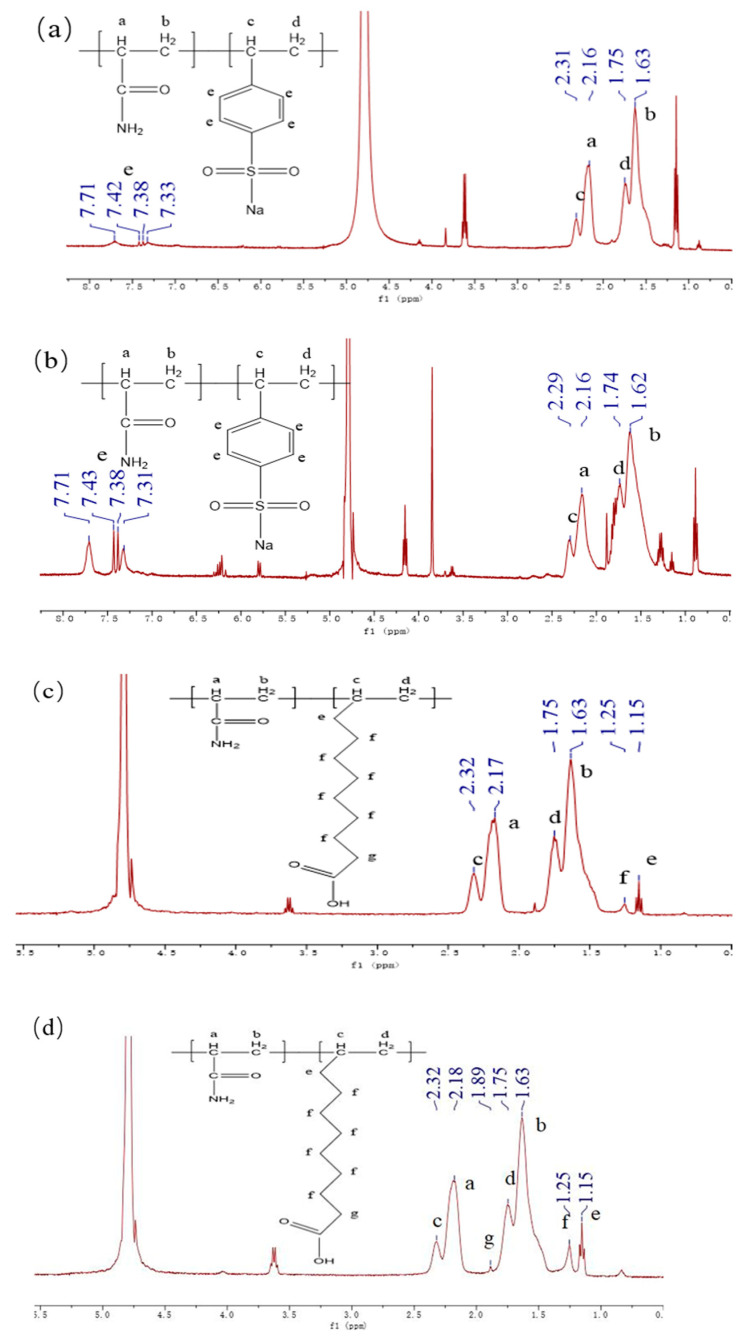
The hydrogen nuclear magnetic resonance spectra of copolymers: (**a**) P(AM-SSS) (H_2_O); (**b**) P(AM-SSS) (ILs); (**c**) P(AM-UA) (H_2_O); and (**d**) P(AM-UA) (ILs).

The hydrogen nuclear magnetic resonance (^1^H NMR) spectra for the P(AM-SSS) copolymer are illustrated in Figure 3a,b. The solvent D_2_O exhibits a proton peak at 4.79 ppm. Within Figure 3a, the proton peaks associated with the methylene groups (-CH_2_, b, d) in the polymer’s main structure appear in the range of 1.63 to 1.75 ppm, while in Figure 3b they are found at 1.62 to 1.74 ppm. Moreover, in Figure 3a the peaks corresponding to the hypomethyl group (-CH, a, c) within the polymer’s main chain are detected at 2.16–2.31 ppm, and in Figure 3b they are recorded at 2.16–2.29 ppm. The benzene ring proton peak (-C_6_H_5_, e), which forms part of the structural unit created by the SSS monomer, is observed at 7.33–7.71 ppm in Figure 3a and at 7.31–7.71 ppm in Figure 3b [30]. In conclusion, the copolymer possesses both AM and SSS linkages.

The hydrogen nuclear magnetic resonance spectrum for the P(AM-UA) copolymer is shown in Figure 3c,d. The proton peak of the solvent D_2_O remains at 4.79 ppm. The peaks in Figure 3c, ranging from 2.18 to 2.32 ppm, and in Figure 3d at 2.17 to 2.32 ppm indicate the presence of protons from the hypomethyl group (-CH, a, c) in the main chain of the polymer. The methylene proton peaks (-CH_2_, b, d) in both figures are positioned at 1.63–1.75 ppm along the polymer’s backbone. Additionally, a peak at 1.15 ppm is linked to the methylene protons (-CH_2_, b, d) in the structural unit derived from the UA monomer, while another peak at 1.25 ppm corresponds to the methylene protons (-CH_2_, f) from a different structural unit that also originates from the UA monomer. Finally, a proton peak at 1.89 ppm is observed for the methylene protons (-CH_2_, g) in yet another structural unit associated with the UA monomer [31]. Thus, the final composition of the copolymer includes both AM and UA linkages.

### 3.2. Monomer Polymerization Rate of Copolymers

Element analysis test results are shown in Table 2. The composition of monomers and their reactivity rates are essential factors for assessing the behavior of copolymerization reactions and the eventual composition of the copolymer produced. To explore how ionic liquids (ILs) as reaction media impact the copolymerization characteristics of various copolymers, reactivity rates for P(AM-SSS) (H_2_O), P(AM-SSS) (ILs), and P(AM-UA) were determined using both the F-R and M-L techniques. The findings, shown in Table 3, indicate that *r*_1_ corresponds to the primary monomer AM, whereas *r*_2_ refers to the comonomer SSS or UA. The analysis demonstrates that employing ILs as reaction media has a noteworthy influence on the reactivity rates of the copolymers. Specifically, the *r*_1_ of P(AM-SSS) increased from 0.2123 (H_2_O) to 0.2752 (ILs), while *r*_2_ decreased from 2.4316 (H_2_O) to 0.1916 (ILs). In contrast, for P(AM-UA) *r*_1_ decreased from 25.5874 (H_2_O) to 10.0131 (ILs), whereas *r*_2_ remained unchanged at 0.0671 (H_2_O and ILs). These findings suggest that while the copolymerization reaction in [BMIM]Oac does not alter the copolymerization type of the monomers, it does influence the tendency for copolymerization by either enhancing or diminishing it.

### 3.3. Sequence Calculation of Copolymer Chain Segments

**Figure 4:**
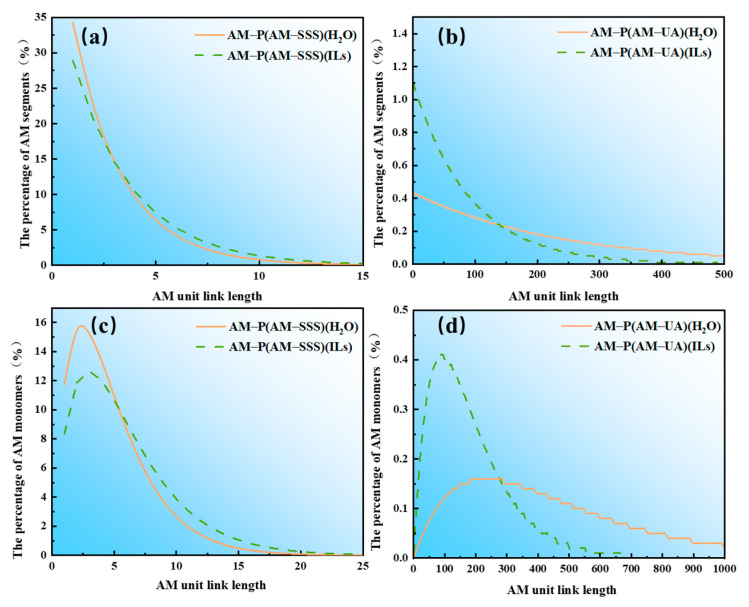
Copolymer chain segment sequence and chain link distribution diagram: (**a**) sequence distribution of P(AM-SSS) copolymer chain segments; (**b**) P(AM-SSS) copolymer chain segment linkage distribution; (**c**) sequence distribution of P(AM-UA) copolymer chain segments; and (**d**) P(AM-UA) copolymer chain segment linkage distribution.

Figure 4 illustrates the distribution of chain segment sequences and linkages for the copolymers P(AM-SSS) and P(AM-UA). As shown in Figure 4a, the copolymer P(AM-SSS), synthesized in aqueous solution, exhibits *F*_1_ = 2.2916, whereas the copolymer synthesized in ILs has *F*_1_ = 3.4043. This indicates that the molecular chain of P(AM-SSS) (H_2_O) comprises a segment with two AM links (hereafter referred to as 2AM, and similarly xAM for consistency) and one SSS link (denoted as 1SSS). In contrast, the copolymer P(AM-SSS) (ILs) predominantly consists of a segment with three AM links (3AM) and one SSS link (1SSS). This finding corresponds to the largest proportion of AM units in the xAM segment relative to the total number of AM units for chain lengths of two (H_2_O) and three (ILs), as depicted in Figure 4b. The average length of the xAM segments in aqueous solution is 2.9170, while in ILs it is 3.4606. When considering the results discussed in Section 3.2 regarding competitive polymerization rates, it becomes evident that the AM monomer copolymerizes with SSS in aqueous solution, resulting in shorter continuous segments of AM units within the polymer molecular chain [31,32]. This suggests that the copolymerization of SSS monomers in water facilitates a more uniform interspersion of AM units within the polymer chain.

Similarly, as illustrated in Figure 4c,d, it is evident that the P(AM-UA) copolymerized in aqueous solution exhibits an *F*_1_ value of 229.5750, while the copolymerization in ILs yields an *F*_1_ value of 90.3589. This suggests that the likelihood of the molecular chain of the copolymer P(AM-UA) (H_2_O) consisting of 229AM and 1UA chain segments is the highest. In contrast, the probability of the copolymer P(AM-UA) (ILs) consisting of 90AM and 1UA chain segments is also the highest. The findings indicate that in aqueous solution the copolymer molecular chain has a lower content of UA monomers and contains a greater proportion of long-chain xAM segments (where x > 140). Conversely, during copolymerization in ILs, the monomers are more uniformly dispersed along the molecular chain, resulting in a significantly reduced presence of long-chain xAM segments (x > 140). Experimental results demonstrate that [BMIM]Oac, as a reaction medium, significantly influences the sequential distribution of the copolymer molecular chain. It effectively dissolves monomers with differing characteristics, thereby addressing the challenges associated with the solubility of hydrophobic monomers and the hydrophilic primary monomer AM. This results in a more homogeneous distribution of hydrophobic monomers within the copolymerization product.

### 3.4. Performance Evaluation of Copolymers

#### 3.4.1. SEM Characterization

**Figure 5:**
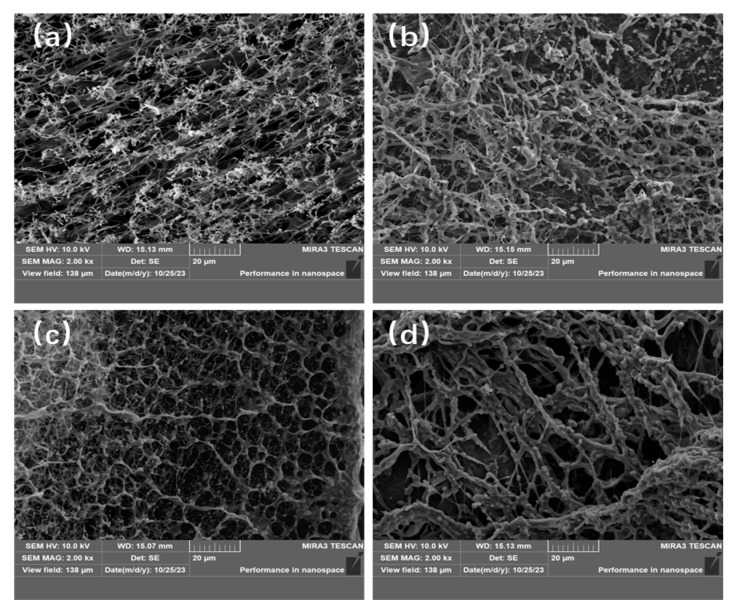
SEM images of the four copolymers: (**a**) P(AM-SSS) (H_2_O); (**b**) P(AM-SSS) (ILs); (**c**) P(AM-UA) (H_2_O); and (**d**) P(AM-UA) (ILs).

Figure 5 presents the scanning electron microscopy (SEM) images of the four copolymers synthesized in water and ionic liquid, respectively, at a concentration of 1000 mg/L. The images reveal that all four polymer solutions exhibit a three-dimensional network structure. Specifically, the copolymers synthesized in aqueous solution display a denser network structure with finer network lines, whereas those synthesized in ILs exhibit a looser network structure with coarser lines. This difference may be attributed to the fact that the products of copolymers synthesized in aqueous solutions are more challenging to remove due to the presence of bound water. The internal network structure of the P(AM-UA) copolymer appears more disordered, likely due to hydrophobic interactions within the molecule. In contrast, the network structure of the P(AM-SSS) copolymer is more hierarchical. But different structures can enhance the performance advantages of copolymers by acting differently [33].

#### 3.4.2. TGA Analysis

**Figure 6:**
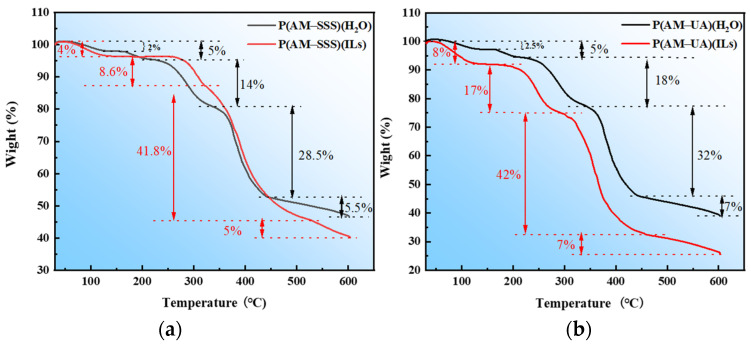
Thermogravimetric curve of the copolymer: (**a**) P(AM-SSS); and (**b**) P(AM-UA).

The thermogravimetric curve for the copolymer P(AM-SSS) is depicted in Figure 6a. Over the complete temperature range examined, the thermogravimetric process can be divided into four unique stages [34]. During the first stage, a weight loss of 5% was observed for P(AM-SSS) (H_2_O) and 4% for P(AM-SSS) (ILs). The mass of the copolymer P(AM-SSS) (H_2_O) initially decreased by 2% within the temperature range of 30–140 °C, after which the mass remained stable until 160 °C. Subsequently, the mass decreased again by 3% in the temperature range of 160–210 °C. This observation indicates that the water bonded to the polymerized structure in aqueous solution is more challenging to remove, necessitating higher temperatures. It is also plausible that P(AM-SSS) (H_2_O) contains a greater number of hydrophilic groups, which further elucidates the phenomenon of higher solid content in the copolymerization products formed in ILs. The second stage for P(AM-SSS) (H_2_O) occurs within the range of 220–345 °C, during which the mass decreases by 14%. In contrast, the second stage for P(AM-SSS) (ILs) takes place between 260 and 340 °C, with a mass decrease of 8.6%. The decrease in mass can be linked to the imide formation from the amide group alongside the thermal breakdown of the amide itself. For P(AM-SSS) (H_2_O) the third stage is detected between 345 and 445 °C, leading to a mass reduction of 28.5%. In contrast, for P(AM-SSS) (ILs) the third stage takes place within 340–520 °C, showing a mass decline of 41.8%. This substantial loss in weight is mainly attributed to the breakdown of the primary chain and the degradation of certain side chains. The fourth stage for P(AM-SSS) (H_2_O) is observed within the temperature range of 445–600 °C, where a mass decrease of 5.5% is recorded. Similarly, the fourth stage for P(AM-SSS) (ILs) occurs from 520 to 600 °C, with a mass decrease of 5%. Comparative analysis indicates that P(AM-SSS) (ILs) exhibits favorable thermal stability.

The thermalgravimetric analysis curve for the copolymer P(AM-UA) is depicted in Figure 6b. Within the examined temperature range, the thermal weight loss process is divided into four unique stages [34]. During the first stage, the observed weight loss was 5% for P(AM-UA) (H_2_O) and 8% for P(AM-UA) (ILs) for the two copolymers. The copolymer P(AM-UA) (H_2_O) initially lost 2.5% of its mass within the temperature interval of 30–130 °C, after which it maintained a constant mass up to 160 °C, followed by an additional mass loss of 2.5% in the 160–205 °C range. This observation suggests that copolymers synthesized in ILs contain a higher amount of adsorbed water and negligible bound water, whereas those synthesized in aqueous solutions exhibit a comparable ratio of adsorbed to bound water. The second phase for P(AM-UA) (H_2_O) occurs between 220 and 340 °C, resulting in an 18% mass decrease, while for P(AM-UA) (ILs) it occurs between 160 and 300 °C, with a 17% mass reduction. Phase three for P(AM-UA) (H_2_O) is noted within the temperature span of 340–445 °C, showing a mass reduction of 32%. In contrast, for P(AM-UA) (ILs) phase three is observed between 300 and 455 °C, reflecting a mass decrease of 42% which is linked to the thermal breakdown of both the polymer backbone and certain segments of the side chains. The fourth stage for P(AM-UA) (H_2_O) is recorded in the temperature range of 445–600 °C, leading to a 7% decrease in mass, while the fourth stage for P(AM-UA) (ILs) also takes place between 455 and 600 °C, exhibiting a comparable 7% mass reduction. These findings indicate that P(AM-UA) (H_2_O) exhibits superior thermal stability compared to P(AM-UA) (ILs).

#### 3.4.3. Evaluation of Increasing Viscosity

**Figure 7:**
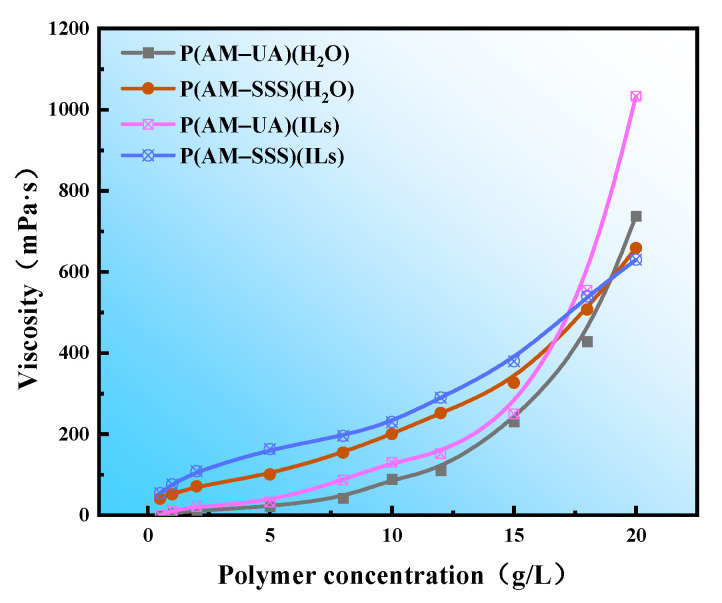
The effect of polymer concentration on its viscosity.

As depicted in Figure 7, IL-synthesized polymers demonstrate enhanced viscosity-concentration dependence compared to their aqueous counterparts. This performance advantage stems from the lower initiator requirements for IL-mediated polymerization, whereas aqueous-phase reactions demand elevated initiator concentrations that may compromise molecular weight. An increase in initiator concentration can lead to a reduction in the molecular weight of the copolymer. In lower concentration solutions the macromolecular chains of the polymer can be fully extended, resulting in only minor differences in apparent viscosity. As the concentration of the polymer rises, the quantity of macromolecules within the system also grows, which boosts the chance of developing a three-dimensional network structure. Moreover, with the increasing concentration of the polymer there is a heightened likelihood of the entanglement of molecular chains, which results in an elevated viscosity of the fluid. In addition, the inclusion of hydrophobic monomers in the polymer chain contributes to a rise in intermolecular hydrophobic interactions as the polymer concentration increases, ultimately leading to the creation of an unstable physical crosslinking network structure. This intricate structure can exhibit improved viscosity characteristics. Consequently, the viscosity of P(AM-SSS), which incorporates hydrophilic monomers, changes relatively gradually with concentration, whereas P(AM-UA), which includes hydrophobic monomers, experiences a marked change in viscosity at a specific threshold known as the hydrophobic aggregation concentration. Notably, P(AM-SSS) demonstrates superior viscosity at lower polymer concentrations (5 g/L).

#### 3.4.4. Evaluation of Temperature Resistance

**Figure 8:**
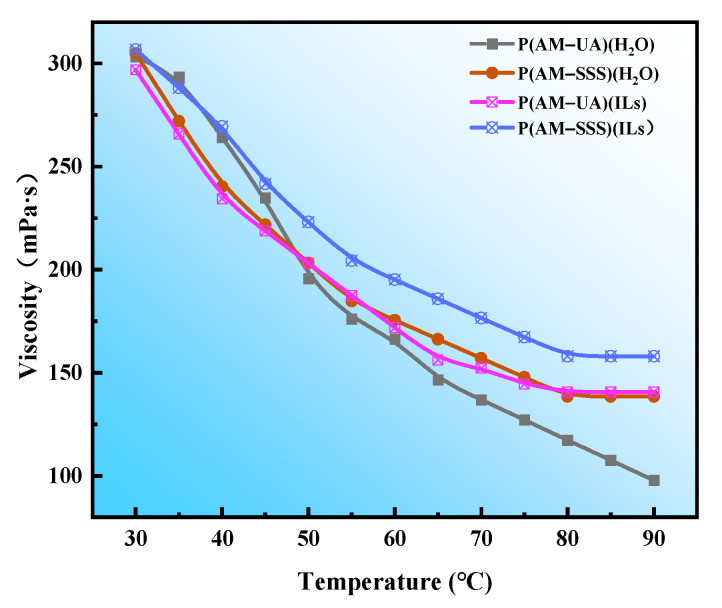
The influence of temperature on the viscosity of polymer solutions.

**Figure 9:**
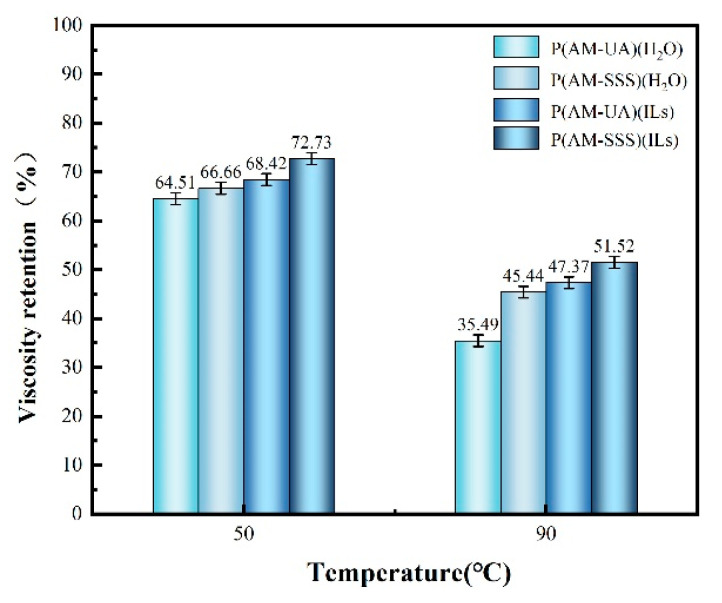
The viscosity retention rate of polymers at different temperatures.

The temperature resistance characteristics of the four synthesized copolymers were evaluated individually, with the findings illustrated in Figure 8 and Figure 9. From Figure 8 it is evident that an increase in temperature leads to a reduction in the viscosity of the polymer solution. This reduction is attributed to the diminished hydration and hydrophobic interactions among the molecular chains of the polymer at higher temperatures, subsequently decreasing the hydrodynamic volume. As a result, there is a noticeable drop in the apparent viscosity of the polymer solution. Figure 9 demonstrates that at a temperature of 50 °C, the viscosity retention of P(AM-SSS) (H_2_O), one of the two copolymers created using an aqueous reaction medium, achieves 66.66%. In contrast, the viscosity retention of the P(AM-SSS) (ILs) polymer, developed with [BMIM]Oac as the reaction medium, reaches 72.73%. When the temperature is raised to 90 °C the viscosity retention for the P(AM-SSS) (H_2_O) polymer is noted at 45.44%, while the P(AM-SSS) (ILs) polymer retains a viscosity of 51.52%. This suggests that both the benzene ring and the sulfonic acid group found in the SSS monomer, along with the extended side chain and significant side group within the hydrophobic UA monomer, play a role in enhancing the rigidity of the polymer chain and improving its resistance to temperature to a certain degree. However, it is important to highlight that the sulfonic acid group not only restricts the hydrolysis of acrylamide but also alleviates the intrinsic limitations of acrylamide’s temperature stability, leading to P(AM-SSS) demonstrating enhanced temperature resistance.

#### 3.4.5. Evaluation of Shear Resistance

**Figure 10:**
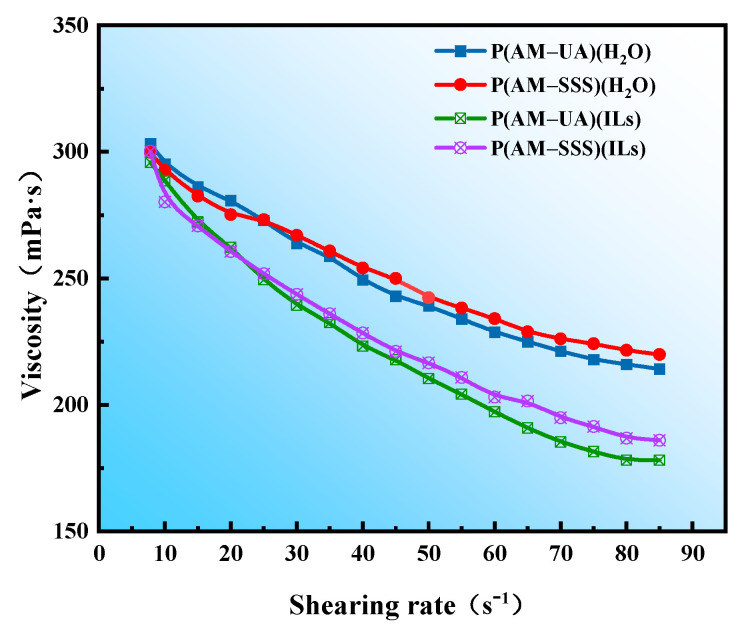
The effect of shear rate on the viscosity of polymer solutions.

**Figure 11:**
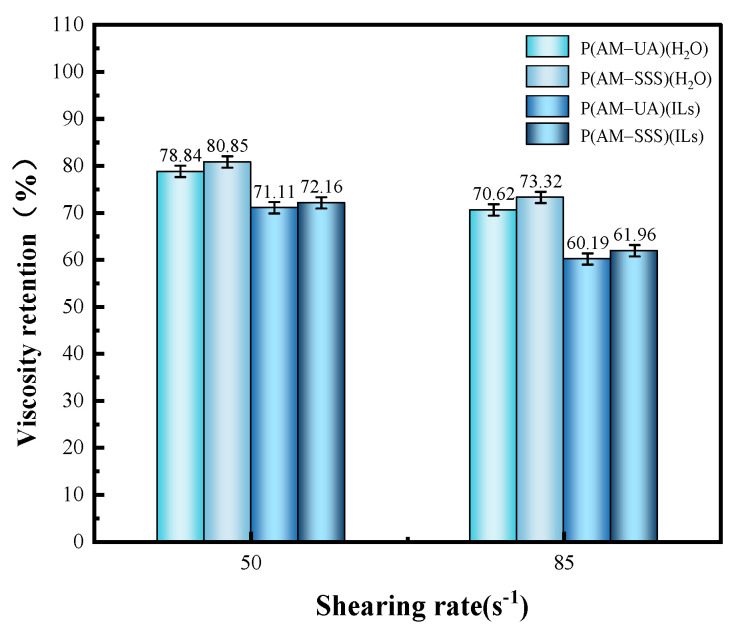
The viscosity retention of polymers at different shear rates.

The evaluation of the four prepared copolymers for shear resistance is shown in Figure 10 and Figure 11. As the results show, the apparent viscosity of polymer solutions declines with an increase in shear rate. Importantly, the shear resistance of the copolymers made in aqueous solution outperforms that of those created in [BMIM]Oac ionic liquid. The microscopic morphology shown in Figure 5 reveals that the copolymer network in water has a denser structure. This closely packed configuration compensates for the less robust network structure, leading to a strong shear resistance in the copolymers synthesized in aqueous conditions. In contrast, the network structure of the copolymers in ionic liquid has thicker lines but is sparse, containing large voids. While this structure may demonstrate considerable resistance at lower shear rates, its efficacy decreases as the shear force intensifies.

#### 3.4.6. Evaluation of Salt Resistance

**Figure 12:**
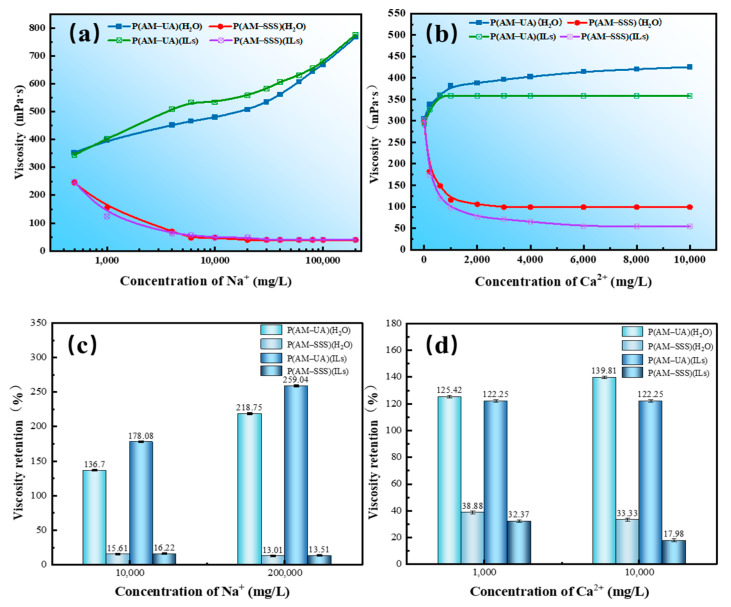
(**a**) The effect of Na^+^ concentration on the viscosity of polymer solutions; (**b**) the effect of Ca^2+^ concentration on the viscosity of polymer solutions; (**c**) the viscosity retention of polymers at different Na^+^ concentrations; (**d**) the viscosity retention of polymers at different Ca^2+^ concentrations.

The four different copolymers were assessed for their resistance to salt, and the findings are illustrated in Figure 12. As indicated in Figure 12a,b, when the concentrations of Na^+^ and Ca^2+^ increased, the apparent viscosities of P(AM-SSS) (H_2_O) and P(AM-SSS) (ILs) showed a decreasing trend. Notably, the viscosity of the P(AM-SSS) (H_2_O) polymer solution remained unchanged as the Ca^2+^ concentration was raised to 2000 mg/L and did not continue to fluctuate with further increases in Ca^2+^ levels. The introduction of Na^+^ and Ca^2+^ ions prompts the previously fully extended macromolecular chains within the polymer solution to start curling, leading to a reduction in the hydrodynamic volume: as salt concentration rises the curling of molecular chains becomes more pronounced [35]. Conversely, the viscosity of the polymer solutions P(AM-UA) (H_2_O) and P(AM-UA) (ILs) rose with increasing concentrations of Na^+^ and Ca^2+^, maintaining high viscosity levels even with Na^+^ concentrations increasing to 10^4^ mg/L and Ca^2+^ concentrations increasing up to 10^3^ mg/L.

From Figure 12c,d, it is apparent that when the Na^+^ concentration reached a significant 10,000 mg/L then P(AM-SSS) suffered a considerable decline in compressive strength, exhibiting viscosity retention rates of merely 15.61% (in H_2_O) and 16.12% (in ILs). Likewise, at a Ca^2+^ concentration of 1000 mg/L the viscosity retention of P(AM-SSS) (H_2_O) and P(AM-SSS) (ILs) fell to 38.88% and 32.37%, respectively. In contrast, P(AM-UA) showcased exceptional salt resistance, revealing that at an extraordinarily high Na^+^ concentration of 200,000 mg/L the viscosity retention rates of P(AM-UA) (H_2_O) and P(AM-UA) (ILs) reached 218.75% and 259.04%, respectively. Even at a Ca^2+^ concentration of 10,000 mg/L, P(AM-UA) (H_2_O) and P(AM-UA) (ILs) upheld impressive viscosity retention rates of 139.81% and 122.25%, underscoring their enhanced resistance to salt.

## 4. Conclusions

Through the investigation of the copolymerization behavior of two copolymer monomers, SSS and UA, with AM in the ionic liquid [BMIM]Oac, the following conclusions can be drawn:

(1) The calculation of polymerization rates for monomers and the sequences of chain segments reveal that the number-average lengths of xAM chain segments in P(AM-SSS) (H_2_O), P(AM-SSS) (ILs), P(AM-UA) (H_2_O), and P(AM-UA) (ILs) are found to be 2.9170, 3.4606, 231.2866, and 91.1179, respectively. This indicates that [BMIM]Oac demonstrates substantial solubility for hydrophobic monomers, facilitating the dissolution of copolymer monomers with diverse characteristics. Consequently, a more homogenous distribution of hydrophobic monomers within the molecular chains of the copolymer products in [BMIM]Oac is achieved.
(2) An analysis of the thermal stability and micro-morphology of the copolymers reveals that the three-dimensional spatial structures of the copolymers synthesized in [BMIM]Oac possess both robust and loose characteristics. The copolymer derived from SSS in ionic liquids displays considerable thermal stability, achieving a decomposition temperature as high as 260 °C, while the copolymer synthesized from UA in water exhibits strong thermal stability with a decomposition temperature of 240 °C.
(3) In comparison to aqueous solutions, the copolymers derived from AM with SSS and UA in ionic liquids show notable thickening properties. Specifically, the copolymer P(AM-SSS) (ILs) exhibits remarkable shear resistance, whereas P(AM-UA) (ILs) showcases exceptional resistance to salt, sustaining a viscosity retention rate exceeding 100% even in high concentrations of Na^+^ and Ca^2+^.

Ionic liquids serve as reaction media to effectively enhance the stability and shear resistance of copolymers [6,22]. Moreover, the ionic liquid [BMIM]Oac exhibits varying improvements in the application performance of copolymers formed from monomers with different solubilities when copolymerized with AM, demonstrating the substantial potential value of ionic liquids that warrants further investigation.

## Figures and Tables

**Table 1:** Monomer feed ratio in copolymer preparation.

P(AM-SSS) (ILs)/P(AM-SSS) (H_2_O)		P(AM-UA) (ILs)/P(AM-UA) (H_2_O)		Monomer Feed Ratio
AM	SSS	AM	UA	
0.0456	0.0051	0.0468	0.0052	9:1
0.0349	0.0087	0.0366	0.0091	8:2
0.0269	0.0115	0.0286	0.0122	7:3
0.0206	0.0137	0.0221	0.0147	6:4
0.0155	0.0155	0.0168	0.0168	5:5

**Table 2:** Element analysis test results.

Copolymer	Reaction Medium	C, wt%	N, wt%	O, wt%	H, wt%	S, wt%
P(AM-SSS)	H_2_O	44.502	7.876	21.063	4.691	8.065
	ILS	40.601	7.796	19.161	4.314	6.697
P(AM-UA)	H_2_O	47.775	18.282	21.079	6.355	/
	ILS	48.500	18.108	21.101	6.561	/

**Table 3:** Calculation results of reactivity of copolymers.

Category	Method	P(AM-SSS)		P(AM-UA)	
		*r*_1_	*r*_2_	*r*_1_	*r*_2_
H_2_O	F-RA	0.2137	2.4645	25.2373	0.0643
	M-L	0.2109	2.3988	25.9375	0.0699
Average value		0.2123	2.4316	25.5874	0.0671
ILs	F-RA	0.2668	0.1938	10.0330	0.0779
	M-L	0.2837	0.1895	9.9932	0.0733
Average value		0.2752	0.1916	10.0131	0.0756

## Data Availability

The original contributions presented in this study are included in the article. Further inquiries can be directed to the corresponding author.
